# Nuances of Responses to Two Sources of Grapevine Leafroll Disease on Pinot Noir Grown in the Field for 17 Years

**DOI:** 10.3390/v14061333

**Published:** 2022-06-18

**Authors:** Jean-Sébastien Reynard, Justine Brodard, Vivian Zufferey, Markus Rienth, Paul Gugerli, Olivier Schumpp, Arnaud G. Blouin

**Affiliations:** 1 Groupe Viticulture, Agroscope, 1009 Pully, Switzerland; jean-sebastien.reynard@agroscope.admin.ch (J.-S.R.); vivian.zufferey@agroscope.admin.ch (V.Z.); 2 Virology-Phytoplasmology Laboratory, Agroscope, 1260 Nyon, Switzerland; justine.brodard@agroscope.admin.ch (J.B.); paul.gugerli@bluewin.ch (P.G.); olivier.schumpp@agroscope.admin.ch (O.S.); 3 Changins College for Viticulture and Oenology, University of Sciences and Art Western Switzerland, 1260 Nyon, Switzerland; markus.rienth@changins.ch

**Keywords:** virome, grapevine leafroll disease, HTS, biological indexing, transmission, *Pseudococcus comstocki*, grapevine asteroid mosaic-associated virus, vitivirus, grapevine leafroll-associated viruses, ampelovirus

## Abstract

Grapevine leafroll disease (GLD) is one of the most economically damaging virus diseases in grapevine, with grapevine leafroll-associated virus 1 (GLRaV-1) and grapevine leafroll-associated virus 3 (GLRaV-3) as the main contributors. This study complements a previously published transcriptomic analysis and compared the impact of two different forms of GLD to a symptomless control treatment: a mildly symptomatic form infected with GLRaV-1 and a severe form with exceptionally early leafroll symptoms (up to six weeks before veraison) infected with GLRaV-1 and GLRaV-3. Vine physiology and fruit composition in 17-year-old Pinot noir vines were measured and a gradient of vigor, yield, and berry quality (sugar content and berry weight) was observed between treatments. Virome composition, confirmed by individual RT-PCR, was compared with biological indexing. Three divergent viromes were recovered, containing between four to seven viruses and two viroids. They included the first detection of grapevine asteroid mosaic-associated virus in Switzerland. This virus did not cause obvious symptoms on the indicators used in biological indexing. Moreover, the presence of grapevine virus B (GVB) did not cause the expected corky bark symptoms on the indicators, thus underlining the important limitations of the biological indexing. Transmission of GLRaV-3 alone or in combination with GVB by *Planococcus comstocki* mealybug did not reproduce the strong symptoms observed on the donor plant infected with a severe form of GLD. This result raises questions about the contribution of each virus to the symptomatology of the plant.

## 1. Introduction

Grapevine (*Vitis vinifera*) has been propagated and distributed throughout the world for centuries since its domestication, which is believed to have taken place about 8000 years ago [1,2]. Viruses have taken advantage of the grapevine vegetative propagation to spread alongside their host. This is best illustrated by the pervasiveness of grapevine rupestris stem pitting-associated virus (GRSPaV), ubiquitous in wine grapes, table grapes, rootstocks, and wild Vitis species, even though this virus has no known vector to date [3,4]. GRSPaV is largely regarded as latent and, as such, it is not included in any virus certification scheme alongside many latent viruses. At the other end of the pathogenicity scale, viruses responsible for the grapevine leafroll disease (GLD) have obvious detrimental effects on wine production. These viruses have been reported in all wine producing regions worldwide. They belong to three distinct genera within the family *Closteroviridae*. The biggest contributors to GLD are grapevine leafroll-associated virus 1 and 3 (GLRaV-1 and GLRaV-3) both belonging to the genus *Ampelovirus*. The name GLD can be attributed to the distinctive symptoms observed in susceptible cultivars after veraison. Additional symptoms include the early reddening of the leaf on red cultivars and a severe reduction in yield [5,6]. Wine quality is also strongly affected due to a general delay in fruit ripening, leading to a decreased level of sugar and anthocyanin contents and increased acidity [7,8]. However, impact of GLD on the grape health and wine quality will depend of the environment, the the age of the plants and how long it has been infected, and the genetics of the players involved, i.e., virus(es), scion, and rootstock [5].

Plant virology has benefited from the tremendous progress in sequencing technologies. High throughput sequencing (HTS) has revolutionized the field of virology by providing an unprecedented tool for the detection and identification of viruses including novel viruses. However, at the same time, virology faces new bottlenecks caused by the imbalance between the large number of viral sequences available and the lack of corresponding biological data [9,10].

Here, to complement a transcriptomic analysis reported previously [8], we report the results on vine physiology and fruit composition from grapevine Pinot noir grafted and infected in the year 2005 with three different inoculums, displaying a range of GLD symptoms, i.e., (1) none (symptomless), (2) mild symptoms infected by GLRaV-1 and named hereafter GLD_S+, (3) and extreme symptoms infected with GLRaV-1 and GLRaV-3 and referred hereafter as GLD_S++. We performed a thorough analysis of the virome from the available Sequence Read Archive (SRA) and a detailed biological characterization. The overall impact of the virus complex on vine performance is discussed.

## 2. Materials and Methods

### 2.1. Plant Material and Experimental Design

The two virus sources used in our experiment were collected in 1984 on two single vines from Pinot noir in a vineyard. The vines showed contrasting symptoms of grapevine leafroll disease (GLD). One plant denoted GLD_S+ showed mild leafroll symptoms expression and was tested positive for the virus GLRaV-1 whereas another showed strong leafroll symptoms (GLD_S++) and was tested positive for GLRaV1, GLRaV-3, and GVB. A symptomless control was obtained by thermotherapy in order to set up a long-term experimental study to evaluate the agronomical and physiological impact of leafroll infection. The materials from the two infected vines together with symptomless control were grafted onto two certified rootstocks: *Vitis berlandieri* × *Vitis riparia* cv. Kober 5BB (referred as 5BB thereafter) and *Vitis riparia* × *Vitis rupestris* cv. 3309 Couderc (referred as 3309C thereafter). The experimental plot was grafted in 2005 and consisted of 10 replicate vines per treatment (symptomless control, GLD_S+ and GLD_S++) per rootstock (5BB, 3309C) for a total of 60 plants. The experiment was conducted at the Agroscope research station in Nyon, Switzerland. The different vines were planted on three adjacent rows in an area with homogeneous soil conditions. Vines were trained in the gobelet training system (or bush vine with shoots vertically attached along one single post) with a planting density of 5500 vines per hectare (0.8 × 1.6 m). Four shoots per vine were maintained.

### 2.2. Vine Physiology and Fruit Composition

Several parameters were assessed during the 6 years (2014–2019). Vine vigor and mortality were assessed on both rootstocks 3309C and 5BB. For gas exchange, fruit composition, and yield, only rootstock 5BB was evaluated. During winters, pruning wood was weighed for each individual plant. Trunk diameters were measured at ca. 10 cm above the soil level. Gas exchange measurements were conducted on two adult non-senescent leaves per plant on four plant per treatment using a LICOR 6400 XT portable photosynthesis system (Lincoln, NE, USA), as described previously [11]. Net photosynthesis, transpiration, and leaf stomatal conductance were measured at saturating light (photon flux density > 1800 mmol m^−2^ s^−1^) on symptomless leaves without virus symptoms when possible. Relative leaf chlorophyll concentrations were measured on mature leaves using a chlorophyll tester called N-tester (Yara International ASA, Oslo, Norway); the measurements (expressed as N-tester units) were taken between June and September. At commercial maturity, grapes were collected and weighed individually for each plant. Grapes were then manually pressed to extract juices, and fruit composition parameters (malic acid, tartaric acid, titratable acidity, pH, Brix, ammonia, and YAN (yeast assimilable nitrogen)) were measured using Fourier transform infrared spectroscopy (WineScan, FOSS NIR Systems, Hillerød, Denmark). Measurements of total anthocyanin content were performed according to the Glories method [12]: briefly, one hundred berries per plant were crushed in a blender, and 50 g of berry skins was extracted at pH 1, and the absorbance was recorded at 280 nm. In 2018 at veraison, sugar content per berry was analyzed by HPLC on a 1260 Infinity Agilent HPLC system, as previously reported [8]. Vine mortality was assessed by observing each plant for any amount of live tissue at the end of the study (fall 2021).

Physiological and fruit composition data were analyzed using R software (https://www.r-project.org/, accessed on 17 June 2022). Statistical analysis was performed by ANOVA followed by mean comparisons using Duncan test from R package Agricolae [13].

### 2.3. Virome Characterization

Biological indexing was performed for the two virus sources and the symptomless control following standard practices for certification [14]. Briefly, five viral diseases were investigated using appropriate indicator varieties: *Vitis vinifera* cv. Gamay, LN33 (1613 Couderc x Thompson Seedless), Kober 5BB, and V. rupestris St. George. Accessions were graft-inoculated onto woody indicators. Symptoms from eight replicates were evaluated in the field over a 3-year period. Positive and negative controls used in the assay were selected from the Nyon grapevine collection [15].

The Illumina sequencing data re-analyzed in this work were obtained from the analysis of a selection of individual berries from the Sequence Read Archive (SRA) database of the National Center for Biotechnology Information (NCBI) under the reference PRJNA594635 [8]. The transcriptomic analysis from which these data were derived were published in 2020. Sequences were obtained from berry samples from GLD_S+ (referred to as T1 in the publication), GLD_S++ (referred to as T2) and symptomless treatments (referred to as C) sampled in August 2017. Following quality trimming, high-quality reads were mapped to the genome of *Vitis vinifera* (PN40024 12X v2). Unmapped reads were de novo assembled and annotated by BlastN and BlastX analysis against reference sequences for viruses and viroids using Geneious v11.1 (https://www.geneious.com/, accessed on 17 June 2022). In the case of GLRaV-1 and grapevine asteroid mosaic-associated virus (GAMaV), contigs were extended by additional rounds of reads mapping. Finally, in order to assess the representation of every virus/viroid in a library, the total number of reads were mapped against the assembled sequences or a similar reference genome from GenBank using the following parameters: medium low resolution and two iterations. For some genome regions of GLRaV-1 and GAMaV, the accuracy of the reconstructed genome was verified by direct Sanger sequencing of PCR products using primers designed from HTS data (data not shown).

Virus status of each of the ten individual plants grafted on 5BB was verified by RT-PCR for the eleven viruses detected by HTS. Two plants from the symptomless treatment were missing as they were removed from the collection at the time of sampling (February 2022). Nucleic acids were extracted from dormant wood with a 3% CTAB buffer (1.4 M NaCL, 20 mM EDTA, 100 mM Tris HCl, and pH8) following publically available protocol (ANSES, 2015). Primers and PCR conditions are presented in Table S1.

All sequences recovered by mapping with a genome coverage (horizontal coverage) greater than 94% were deposited on GenBank under accessions ON221453-ON221466 and ON237610. The sequences of GLRaV-1 and GAMaV obtained from both mapping and RT-PCRs were deposited under accessions ON221467, ON221468, and ON583999 (Table S2).

### 2.4. Mealybug Transmission Experiment

In summer 2017, one individual mealybug *Pseudococcus comstocki* was collected from a plum tree in a commercial orchard in Swiss orchards (Valais region) and a colony was started and maintained on potato tubers (*Solanum tuberosum*) in jars. Given that mealybug species might be difficult to distinguish based on their morphology, DNA barcoding identification was performed: Genomic DNA was extracted using the DNeasy extraction kit (QIAGEN) and a part of the mitochondrial COI gene was amplified and sequenced according to Correa [16]. Virus transmission assays were conducted according to Le Maguet et al. [17]. Briefly, during the acquisition access period, leaves of donor vines were collected in field during autumn 2018 and were kept in jars containing mealybugs. Following a 72-hour acquisition-access period, batches of ca. 10 mealybugs (instar nymphs L1 and L2) were transferred onto a recipient plant for the inoculation access period (IAP). The recipient plants were cuttings of healthy vine of Pinot noir kept in pots in a greenhouse for the duration of the trial. After 72 h of IAP, the inoculated plants were sprayed with an insecticide. The transmission test was made with eight recipient vines. Recipient vines were maintained at 20 °C and 16/8 h (light/dark) under artificial light. Viral infections in recipient plants were assessed by RT-PCR, eight months post inoculation, and included dormancy period. Recipient plants were kept in a greenhouse and leafroll symptom expression was recorded in autumn at twelve months post-inoculation.

## 3. Results

### 3.1. Symptoms Development, Agronomical Impact and Effects on Fruit Composition

**Figure 1:**
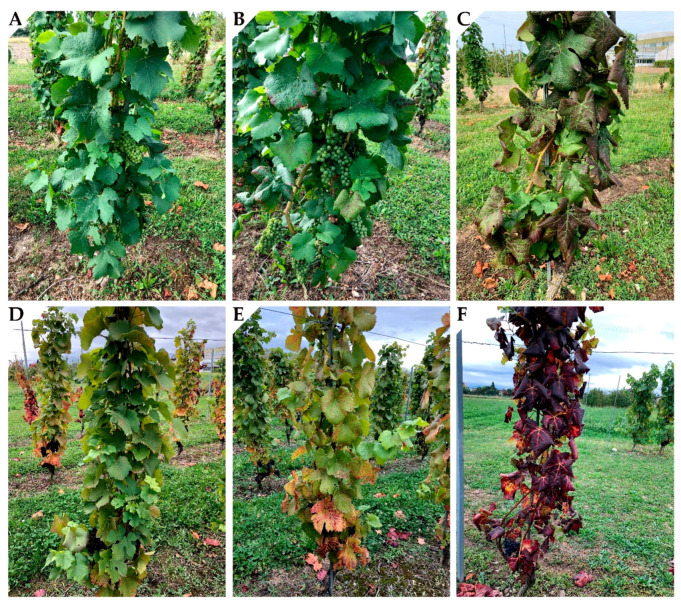
Symptomatology displayed on the three treatments of *Vitis vinifera* cv. Pinot noir on rootstock 5BB. Before veraison, early August (**A**–**C**) and at harvest in early October (**D**–**F**). Symptomless control with no leafroll symptoms (**A**,**D**); GLD_S+: grapevine leafroll disease causing mild symptoms (**B**,**E**); GLD_S++: grapevine leafroll disease causing severe symptoms (**C**,**F**).

Symptoms observed in source vines were reproducible in our field experiment (Figure 1). Visual symptoms appeared for GLD_S+ treatment in early August and consisted in interveinal reddening without apparent downward leaf rolling (Figure 1). For treatment GLD_S++, symptoms (leaf reddening) appeared from mid of June on, about six weeks pre-veraison (Figure 1C). At veraison, GLD_S++ showed leaf reddening and downward leaf rolling affecting most of the canopy (Figure 1). At harvest in treatment GLR_S++, symptoms affected the entire canopy area toward the shoot apex. At the end of the growing season (October–November), leaf senescence occurred 2–3 weeks earlier in treatment GLR_S++. These symptoms were consistent every year.

**Figure 2:**
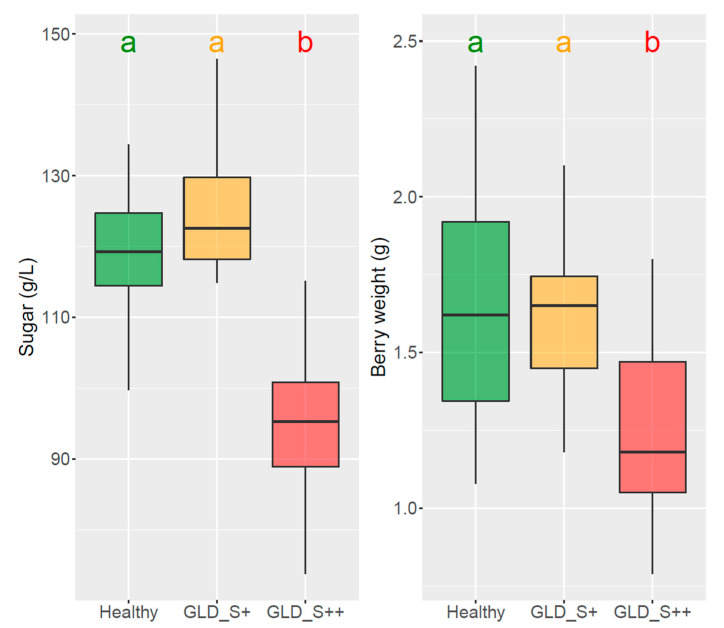
Single berry sugar content and berry weight and at veraison (14.8.18) in *Vitis vinifera* cv. Pinot noir on rootstock 5BB infected or not by leafroll disease. Means were denoted by different letters (a,b) when they differ significantly at *p* < 0.001 using Ducan’s new multiple range test. N = Healthy (symptomless): 31; GLD_S+: 15; GLD_S++: 25. GLD_S+: Grapevine leafroll disease causing mild symptoms; GLD_S++: grapevine leafroll disease causing severe symptoms.

In 2018, sugar content and weight were assessed in single berries at the onset of veraison (when first visible signs of color change occurred). Sugar was significantly lower for the modality GLD_S++ when compared to the symptomless control or to GLD_S+. This sugar drop was not observed between GLD_S+ and the control (Figure 2).

**Figure 3:**
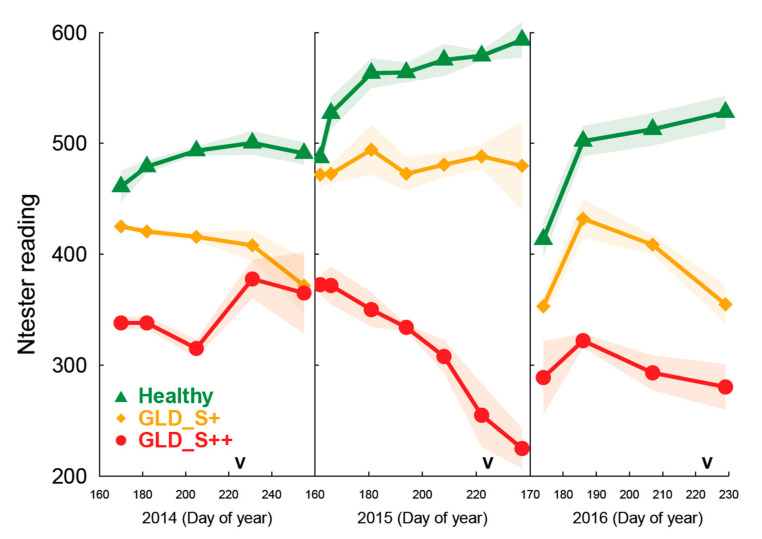
Evolution of leaf chlorophyll content (N-tester reading) during three consecutive growing seasons in *Vitis vinifera* cv. Pinot noir on rootstock 5BB infected or not by leafroll disease. Healthy: symptomless; GLD_S+: grapevine leafroll disease causing mild symptoms; GLD_S++: grapevine leafroll disease causing severe symptoms. Letter V represent date of veraison (i.e., change of fruit color from green to blue). Data are expressed as the mean ± standard deviation (shading).

The seasonal evolution of leaf chlorophyll content is shown in Figure 3. Compared to symptomless control, treatment GLD_S++ showed a drastic reduction in leaf chlorophyll content and this was already observable in mid-June. The chlorophyll content of the GLDS+ treatment leaves always remained between that of the symptomless control and that of GLD_S++. For both treatments, a significant reduction in leaf chlorophyll content could be measured even before the appearance of visible symptoms. These observations were consistent over the three years of measurements. In particular, during 2015, leaf chlorophyll content for symptomless control increased from June to August (veraison). On the contrary, leaf chlorophyll content for GLD_S++ was constantly diminishing during the same period and it reached 33% of the leaf chlorophyll content measured from the symptomless control at veraison while the level remained flat during the same period for GLD_S+.

**Figure 4:**
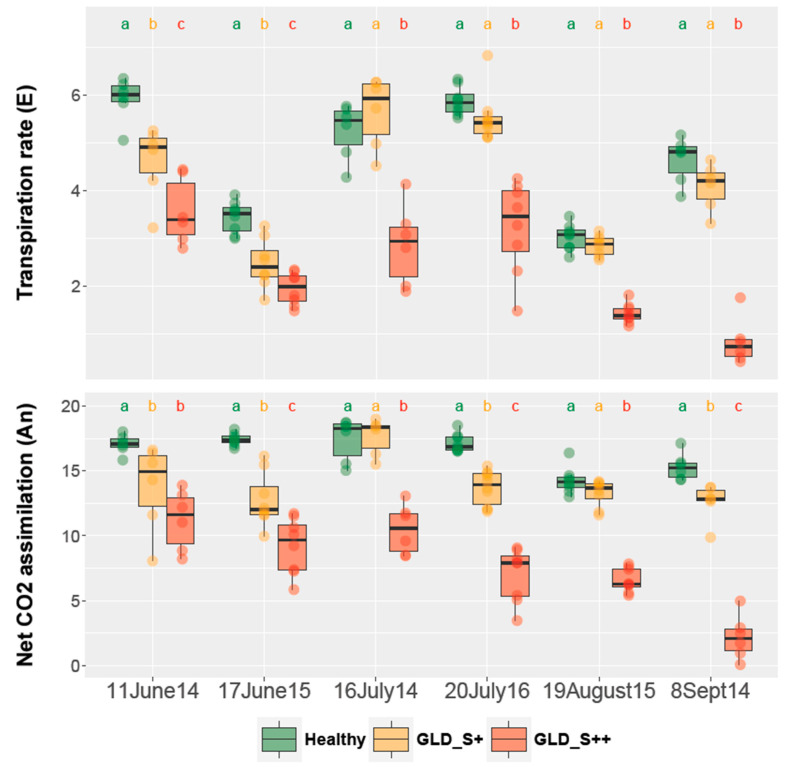
Effect of grapevine leafroll disease on gas exchange parameters in *Vitis vinifera* cv. Pinot noir on rootstock 5BB. Transpiration rate is expressed as mmol H_2_O m^−2^ s^−1^, net photosynthesis (AN) as µmol CO_2_ m^−2^ s^−1^. Measurements were recorded on six different days over three seasons. Means were denoted by different letters (a, b, and c) when they differ significantly at *p* < 0.05 using Ducan’s new multiple range test. N = 8 per treatment and date. Healthy: symptomless; GLD_S+: grapevine leafroll disease causing mild symptoms; GLD_S++: grapevine leafroll disease causing severe symptoms.

Net photosynthesis (AN) was assessed by measurements of gas exchange, transpiration and stomatal conductance over six days during three seasons (Figure 4). In leaves from symptomless controls, AN was relatively stable with values between 13 and 18 µmol m^−2^ s^−1^ over the three seasons, from June to September. All measurements on GLD_S++ leaves showed a significant reduction in AN and transpiration rate (Figure 4). This reduction on GLD_S++ leaves was further exacerbated in September and reached 1/5th of the value of the symptomless control. Similar trend was observed for stomatal conductance (data not shown). For GLD_S+ treatments, the effect on gas exchange was less pronounced. However, a significant reduction was observed for AN in three out of six dates; a similar drop was measured for the transpiration rate in two out of six dates. GLD_S+ treatment reached a maximum reduction of 30% for AN and transpiration rates compared to symptomless control plants (17 June 2015).

Vegetative growth was evaluated by measuring pruning weights and trunk diameter based on rootstock and viral status. Statistical analysis indicated that there was no significant viral treatment x rootstock interaction for vine vigor. However, we observed a tendency of a stronger effect on vine vigor for the treatment GLD_S++ on rootstock 3309C compared to 5BB (Table 1 and Table 2). Pruning weights were strongly affected in treatment GLD_S++, with a significant reduction of, on average, 55% and 78% on rootstock 5BB and 3309C, respectively (Table 1). For treatment GLD_S+, average pruning weights were lower over the four seasons compared to the symptomless control. However, the 9 to 20% decrease in pruning weights was not statistically significant every season and was not dependent on rootstock.

For trunk diameter and mortality evaluated at the end of the study, the data showed a more severe effect for GLD_S++ on rootstock 3309C compared to 5BB (Table 2). In GLD_S++ vines, the diameter of the trunk was reduced by 31% on 5BB, while it reduced by 41% on 3309C rootstock. All vines grafted on 5BB survived until the end of the experiment (Table 2). On contrary, 4 out of 10 vines from GLD_S++ on 3309C died during the experiment. Trunk diameter was significantly smaller in GLD_S+ compared to the symptomless control (ca. 10% reduction) for both rootstocks. For treatment GLD_S+, all vines on both rootstocks survived until the end of the experiment.

Fruit composition and yield at harvest were affected in each of the three seasons by leafroll disease (Table 3). Treatment GLD_S++ was associated with a severe decrease (ca. 80%) in yield per vine. For GLD_S+, no effect on yield was observed for season 2015, 2016. However, a 50% reduction was recorded in 2017 (Table 3). In 2016, juices from GLD_S++ vines were significantly lower in total soluble solids. In 2015, tartaric acid content and total acidity were significantly lower in berries from GLD_S++. Finally, berry nitrogen concentration (YAN) was consistently affected by leafroll disease over the three harvests. GLD_S+ reduced berry nitrogen concentration by an average of 30%. On the other hand, GLD_S++ treatment implied a YAN reduction of nearly 70%. Finally, the amounts of total berry anthocyanins measured in 2016 and 2017 were not significantly different between the different treatments (data not shown).

### 3.2. Virome Characterization

**Figure 5:**
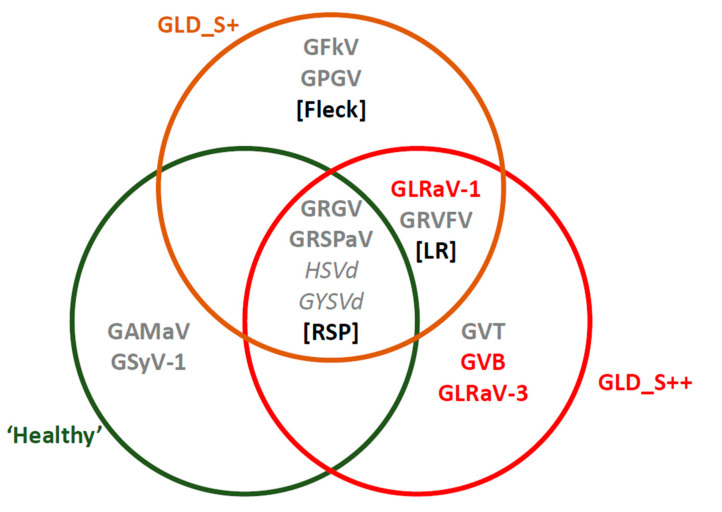
Representation of the viruses detected by High throughput sequencing and pathogen detected by biological indexing in different group (‘healthy’ (symptomless) in green, GLD_S+ in brown and GLRD_S++ in red). Colour of the virus front based on the expected pathogenicity: background (grey) or pathogen (red). Biological indexing positive detection are marked in black in []. LR is for leafroll disease observed on *Vitis vinifera* ‘Gamay’. Fleck is for fleck detection on *Vitis rupestris* ‘Saint Georges’. RSP is for Rupestris stem pitting detection on *Vitis rupestris* ‘Saint Georges’. Corky bark (CB) on LN33 (1613 Couderc x Thompson Seedless) and Kober stem-grooving (KSG) on Kober 5BB were also tested but all the treatments returned negative. Virus acronyms: GRSPaV—grapevine rupestris stem pitting-associated virus; GRGV—grapevine red globe virus; GAMaV—grapevine asteroid mosaic-associated virus; GSyV-1—grapevine Syrah virus 1; GFkV—grapevine fleck virus; GPGV—grapevine Pinot gris virus; GLRaV-1—grapevine leafroll-associated virus 1; GLRaV-3—grapevine leafroll-associated virus 3; GRVFV—grapevine rupestris vein feathering virus; GVT—grapevine virus T; GVB—grapevine virus B.

The viromes of both leafroll disease sources GLD_S+, GLD_S++, and symptomless control were characterized simultaneously using both the biological indexing assay and high-throughput sequencing (HTS) analysis. For the bioassay, disease symptoms on indicators were evaluated during three seasons after graft inoculation. The presence of rupestris stem pitting was visible on the three treatments, with pitting and grooving observed under the bark of the *Vitis rupestris* St-George indicator. This was the only disease detected from the symptomless control. Leafroll symptoms were recorded on samples GLD_S+ and GLD_S++ with early reddening and rolling of the Gamay indicator. Lastly, the fleck symptoms were recorded in the GLD_S+ samples on the indicator *Vitis rupestris* St-George, with a decreased vigor and vein clearing (Figure 5). The SRA of berry tissues obtained for each treatment between ca. 136 and 160 million reads in total when combining the three technical replicates and the two time points. The de novo assembly allowed the identification of the viruses present. For each of the virus/viroid identified in the dataset, a mapping of the reads against reference sequences was performed. The numbers of mapped reads and genome coverage are shown in Table S2 for every identified virus/viroid. A small fraction of the reads was virus/viroid-derived, and host-derived sequences accounted for the majority of reads, with 0.56 M viral reads obtained from symptomless (0.35%), 0.97 M viral reads from GLD_S+ (0.67%), and 2.51 M viral reads from GLD_S++ (1.84%). In total, eleven plant viruses and two viroids were detected. The two viroids, hop stunt viroid (HSVd) and grapevine yellow speckle viroid 1 (GYSVd1), and the two viruses, grapevine rupestris stem pitting-associated virus (GRSPaV) and grapevine red globe virus (GRGV), were present in all samples. Two additional viruses were only detected in the symptomless treatment: GAMaV and grapevine Syrah virus-1 (GSyV-1). Six viruses were detected in the GLD_S+ treatment, including grapevine fleck virus (GFkV) and grapevine Pinot gris virus (GPGV) that were only found in this treatment, and two, GLRaV-1 and grapevine rupestris vein feathering virus (GRVFV), that were also present in GLD_S++. Lastly, a total of seven viruses and two viroids composed GLD_S++ virome. Grapevine virus T (GVT), grapevine virus B (GVB), and GLRaV-3 were unique to this treatment (Figure 5).

The presence of these viral species derived from the SRA was confirmed using specific RT-PCR analysis on each individual vine, and the results are presented in Table S1. The main virus infections were found to be present in all ten plants of each treatment, in accordance with SRA data (GLRaV-1, GLRaV-3, GVB, GVT, GRSPaV, and GRVFV). GRGV was detected in all but five plants (two GLD_S++ and three symptomless), and GSyV-1 and GAMaV were detected in six and two of the eight symptomless plants tested, respectively. GPGV was only detected in 2 of the 10 GLD_+, but it was also found in 2 of the 10 GLD_S++ plants where it was not detected from SRA.

### 3.3. Mealybug Transmission Experiment

**Figure 6:**
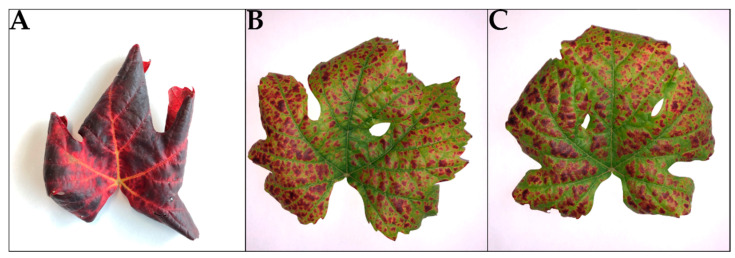
Transmission experiment using *Pseudococcus comstocki* to disentangle mixed viral infection. Original viral source on Pinot noir was displaying very severe symptoms of leafroll disease (**A**), whereas mealybug transmitted grapevine leafroll-associated virus 3 (GLRaV-3) (**B**) and mixed-infection GLRaV-3 grapevine virus B (**C**) on Pinot noir were causing milder leafroll symptoms.

In an attempt to disentangle the different viruses present in GLD_S++, mealybug transmissions were performed. Eight recipient Pinot noir plants were screened for GLRaV-1, GLRaV-3, and GVB infection. Healthy control vines used in transmission assay tested negative for all viruses. Three recipient plants were found to be infected with GLRaV-3 including one mixed-infection with GLRaV-3 and GVB. No virus was transmitted in the remaining five recipient plants. GLRaV-1 was not detected. There was no difference in symptoms expression between the plant mixed-infected with GLRaV-3 and GVB and the two other infected by GLRaV-3. Furthermore, symptoms on these three plants were less severe than the original viral source GLD_S++ (Figure 6).

## 4. Discussion

In this study, we examined the impact of leafroll disease on 17-year-old field-grown pinot noir in complement to the previously published transcriptomic analyses. The inoculum GLD_S++ was selected in this experiment because it consistently displayed one of the strongest leafroll symptoms amongst about 900 virus-infected Vitis accessions held in the Agroscope collection, of which at least a third contained one of the five main GLRaVs from different origins present in single or multiple infection [15]. The GLD_S+ inoculum was selected for showing mild but characteristic leafroll symptoms. In light of the viral composition observed here, it cannot be excluded that some of the viruses detected in the symptomless control altered the physiology of the plants. However, these controls showed no viral symptoms and behaved as would be expected for healthy Pinot noir in this environment.

These radical differences in symptomology were reflected by three highly distinct viromes, only sharing the two viroids (HSVd and GYSVd) and two viruses (GRSPaV and GRGV). However, with the notable exception of GLRaV-1, strong genetic variability was observed within species sequences obtained from different treatments (GRSPaV, GRGV, and GRVFV). It is important to note, however, that these sequences were assembled from a pool of plants and only the dominant sequence or sequences were recovered. Virome homogeneity within a treatment was confirmed by RT-PCR on individual plants with the exception of GPGV and GAMaV only detected on a few plants and, to a lesser extent, GRGV and GSyV-1 detected in most but not all plants (Table S1). From the 11 viruses identified, only GLRaV-1, GLRaV-3, and GVB would be considered as candidates for causing the observed symptoms. Yet, it would be risky to assume that the remaining (highly different) viral background has no effect on the dramatic physiological alteration measured.

Early interveinal reddening of the leaves observed on the red cultivars is the first symptom of GLD, followed by downward leaf rolling. It is interesting to note that GLD_S++-infected Pinot noir plants were consistently the first vines of the Agroscope collection to express those leafroll symptoms and more intriguing is that symptoms were observed as early as six weeks prior to veraison. Leafroll-infected plants are known to only express symptoms after veraison and that the timing of the symptom is not only dependent on the inoculation time but also the virus strain in the case of GLRaV-3 infections [18,19]. Plants in this study have been infected for almost 20 years, and the grapevine leafroll-associated viruses isolates are very similar to those present on GenBank, and they are not expected to produce unfamiliar symptoms. GLRaV-1 and GLRaV-3 genotypes detected are within the main cluster (group I) of recent phylogenetic analyses for both genera [20,21]. Both treatments have an almost identical GLRaV-1 genome (99.6% id). Furthermore, when untangled by mealybug inoculation, the addition of GVB to GLRaV-3 did not worsen the symptoms of Pinot noir (Figure 5) and both symptoms caused by GLRaV-3 alone, or with GVB, produced symptoms comparable to those observed on GLD_S+. Albeit serendipitous, this constitutes the first evidence of GVB transmission by *P. comstocki*. This similarity of symptoms suggests that the severe symptomology of GLD_S++ is caused by a synergy between GLRaV-1 and GLRaV-3 with a possible effect of GVB. However, a specific interaction between the viruses and the rootstock (here 3309C or 5BB) cannot be ruled out, since the recipient Pinot noir plants in mealybug transmission were own-rooted (no rootstock).

Similarly, we cannot exclude the impact of less studied viruses such as GVT for which its presence is much more likely be unnoticed, in addition to a known pathogen (GLRaV-3). In this respect, it is interesting to note that the increase in the number of virus species detected per treatment (four in symptomless, six in GLD_S+, and seven in GLD_S++) was reflected by an increased percentage of virus reads in total sequencing, estimated around 0.35% for the symptomless treatment, 0.67% for GLD_S+, and 1.84% in GLD_S++ (Table S2). Moreover, for the same virus infection, GRGV, the read number was increased more than 10-fold between the symptomless and GLD_S+ and another 10-fold between GLD_S+ and GLD_S++ (from 2 reads mapped per million sequenced in the symptomless to 25 in GLD_S+ to 295 in the GLD_S++). This apparent enhanced environment for virus replication in the plant GLD_S++ (at least in the berries) could be caused by a strong RNA silencing suppressor expressed from grapevine leafroll-associated virus 3 [22,23]. The role of the VSRs in the synergy between viruses is well described in different pathosystems reviewed in [24]. In grapevine, increased vitivirus loads were measured in plants co-infected by a member of closterovirids [25]. In our GLD_S++ data, the total number of GVB reads represented more than 62% of all viral reads. This increased vitivirus titer may be the reason why vitivirus transmission is facilitated when the donor plant is also infected by a leafroll virus [26,27,28]. Virus distribution will vary between generative and vegetative organs as well as with respect to the berry developmental stage, and this analysis only relied on sequencing data of veraison and mid-ripening berries. In contrast to the high vitivirus load measured, the low number of reads of the ampeloviruses GLRaV-1 and GLRaV-3 is probably due to the phloem-limited nature of the viruses and the fact that RNA extraction was made on berries just at the beginning of the veraison before the phloem flushed to the berry [29].

Previously, transcriptome analysis of those plants had shown an upregulation of the plant-innate immunity [8]. The observed phenological shift and the visible impact of GLD on vine physiology were confirmed by the present analysis, specifically regarding berry weight, sugar accumulation, chlorophyll content, transpiration, gas exchange, yield, and vigor. For all those metrics, the performances of vines from treatment GLD_S+ were much more similar to those of the symptomless control as compared to GLD_S++, confirming, the conclusion drawn from berry transcriptomic analyses at the scale of the plant physiology [8]. Despite no significant difference between the control and GLD_S+ for the berry’s size and sugar accumulation, a moderate impact on photosynthesis parameters was recorded that did not clearly translate into a deterioration of fruit composition. Yield and vigor were affected in less than half of the monitored years (one out of three yield measurements and one out of four measured pruning weights for the rootstock 5BB). This makes the virome composition of GLD_S+ a mild virus cocktail given the pedoclimatic conditions. Conflicting results were described on Nebbiolo infected with GRLaV-1 and GVA with one study showing no significant difference in yield and brix compared with non-infected control vines [30], where another study reported a drop in yield under their conditions [31], which confirms the significant importance of environmental parameters but perhaps also of the fine genetic components in the agronomic impact of strains or viral cocktails considered to be of low virulence.

On the other hands, all the assessed parameters from GLD_S++ were severely altered compared to the control (yield, vigor, sugar accumulation, and photosynthesis parameters). The lower sugar content and berry weight indicate a delay in the onset of veraison for the severe form of GLD; thus, a general delay in berry development is often reported in GLD-infected vines [32]. The negative effect of GLRaV-3 (presumably alone) on yield was already clearly demonstrated on red cultivars [7,33,34], and the impact on the photosynthesis was also well described before [34,35]. The GLS_S++-infected Pinot noir not only had a much-reduced yield with less than 20% of the harvested weight of the control, but the berry’s quality was not up to winemaking standards with a drastic drop of YAN, which only accounted for 1/3 of the control and is a level that is detrimental for winemaking [36]. The detrimental effects of GLD on YAN were measured before on GLRaV-2 and GLRaV-3 grapevine-infections but not to such drastic levels [37]. Lastly, vine vigor was extremely affected as well. Vigor reduction is often associated with GLD infection, especially with GLRaV-3 [5,6]. The impact on plant vigor is probably only visible several years after infection due to a slow depletion of reserve due to lower AN. The effects on yield and sugar accumulation are measurable earlier, as demonstrated by Cabaleiro who compared infected and non-infected vines with similar vigor [38]. Overall, our data suggest that the inoculum selected 17 years ago for this project is showing a gradient response to viral infections from the symptomless group to GLD_S+ and GLD_S++.

Although most of the physiological data were obtained from the rootstock 5BB, vigor and mortality were measured on 3309C and showed an increased disease severity. The rootstock influence on symptoms severity is well documented [38,39,40,41,42,43,44]. Our data confirmed previous research showing that Pinot noir on 5BB rootstock could be more vigorous than on 3309C in some conditions [45]. The 40% mortality observed on treatment GLD_S++ on the rootstock 3309C resembles, to some extent, those of a leafroll source (LR132) from US Davis viral collection responsible for vine death when inoculated on Cabernet franc propagated on 3309C but not when the rootstock used was 5BB [44]. The inoculum source of LR132 was composed of a GLRaV-1 and GVA. Our GLD_S++ was composed, amongst other, of GLRaV-1, GLRaV-3, and GVB.

Finally, the strength of HTS for plant diagnostic was exposed when we compared the virome identified by sequencing to the results of a three-year long biological indexing assay (Figure 5). The biological assay is commonly used in wine-producing countries to detect viral diseases. In Switzerland and EU, this approach is the official method to admit a clone in certification programs [46,47,48,49]. When compared with HTS, the biological indexing missed the detection of two viruses that were expected to be detected. GAMaV identified by HTS did not induce symptoms on the indicator V. rupestris but its presence was confirmed by RT-PCR in the original plants and in the V. rupestris indicator (data not shown). Biological information on this virus and. to some extent, to most grapevine-infecting tymovirids is sparse, suggesting that they are of little concern to the wine industry. Very few evidence of GAMaV detection by indexing has been published [50,51]. This result constitutes, incidentally, the first report of GAMaV in Switzerland (accession ON583999). The second discrepancy between the HTS data and the biological indexing is a greater source of concern as it is GVB not causing corky bark diseases on LN33. In contrast to previous reports of GVB isolates not producing corky bark symptoms [52], the detection here of two very distinct GVB genotypes in GLD_S++ plants downplays the theory of a non-symptomatic isolate. On the contrary, the overall stress exerted by the virome on the indicator clearly reduced its growth and, thus, probably annihilated the symptoms. In contrast to previous reports of GVB isolates not producing corky bark symptoms [52], the detection here of two very distinct GVB genotypes in GLD_S++ plants refutes the theory of a non-symptomatic isolate. Instead, the overall stress exerted by the virome on the indicator clearly reduced its growth and thus probably annihilated the symptoms. GVB is regulated and its role of some of its variant in the graft incompatibility and decline is documented [25,53]. The advantage of HTS over biological indexing for virus detection was not only previously presented for grapevine but also for fruit trees [54,55] and our research backs the drive toward a modification of the certification programs to include HTS under internationally recognized guidelines [56,57].

## 5. Conclusions

To conclude, our study confirmed the serious impact caused by GLD on the plant fitness and wine quality and that severity can be altered by the rootstock selected. In all quantitative and qualitative measurements, GLD_S+ vines showed an intermediate response to the disease between symptomless and GLD_S++, making it a good gradient model for the virus response, but the complexity of the viromes identified did not allow the severity of symptoms to be attributed to the addition of a single virus but rather to a synergy between the viruses present. This research reports the presence of GAMaV for the first time in Switzerland and the first transmission of GVB by *P. comstocki*. Finally, this study confirms the strength of HTS compared to biological indexing with a faster and more sensitive virus detection. It is important to make good use of this technology to ensure the sanitary status of the planting material as it is, together with the vector control, our only guarantee for a healthy vineyard.

## Figures and Tables

**Table 1:** Effect of rootstock and virus status on vine vigor (weight of pruning wood (g/vine)) on *V. vinifera* cv. Pinot noir over four consecutive years. GLD_S+: Grapevine leafroll disease causing mild symptoms; GLD_S++: grapevine leafroll disease causing severe symptoms. Means were denoted by different letters (a, b, and c) when they differ significantly at *p* < 0.05 using Ducan’s new multiple range test.

	2015				2016				2017				2018			
	Rootsock															
Treatment	5BB	% *	3309C	% *	5BB	% *	3309C	% *	5BB	% *	3309C	% *	5BB	% *	3309C	% *
Symptomless	460 a		338 a		480 a		305 a		391 a		220 a		540 a		399 a	
GLD_+	403 a	−12	296 b	−12	380 b	−20	263 a	−13	295 b	−20	195 a	−12	490 a	−9	349 a	−12
GLD_S++	199 b	−43	64 c	−81	211 c	−56	54 b	−83	159 c	−59	64 b	−62	205 b	−62	88 b	−78

* Percent decrease relative to symptomless control.

**Table 2:** Mortality rate (dead vines/total number of vines planted at the study’s beginning) and trunk diameter (mean) measured at the end of the experiment according to rootstock and virus infection. Means followed by different letters differ significantly at *p* < 0.01 by Ducan’s new multiple range test. GLD_S+: Grapevine leafroll disease causing mild symptoms; GLD_S++: grapevine leafroll disease causing severe symptoms. Means were denoted by different letters (a, b, and c) when they differ significantly at *p* < 0.05 using Ducan’s new multiple range test.

Rootstock						
Treatment		5BB			3309C	
	Mortality	Trunk Diameter (cm)	% *	Mortality	Trunk Diameter (cm)	% *
Symptomless	0/10	3.5 a		0/10	3.1 a	
GLD_S+	0/10	3.2 b	−9	0/10	2.8 b	−10
GLD_S++	0/10	2.4 c	−31	4/10	1.8 c	−42

* Percent decrease relative to the symptomless control.

**Table 3:** Impact of grapevine leafroll disease on yield and fruit composition of *V. vinifera* cv. Pinot noir on rootstock 5BB over three consecutive years. Means within year followed by different letters differ significantly at *p* < 0.05 by Ducan’s new multiple range test. GLD_S+: Grapevine leafroll disease causing mild symptoms; GLD_S++: grapevine leafroll disease causing severe symptoms. Total (titratable) acidity is expressed in g/L of tartaric acid. YAN: yeast assimilable nitrogen (mg/L); n.a.: data not taken.

	Yield (kg/vine)	Soluble Solids (°Brix)	pH	Total Acidity	Tartaric Acid (g/L)	YAN
**2015**						
Symptomless	0.6 a	22.2 a	3.12 a	8.6 a	7.9 a	178 a
GLD_S+	0.5 a	22.5 a	3.10 a	8.3 a	7.5 a	132 a
GLD_S++	0.1 b	22.4 a	3.33 b	6.8 b	6.4 b	65 b
**2016**						
Symptomless	1.0 a	24.1 a	3.23 a	8.0 a	6.4 a	151 a
GLD_S+	1.1 a	23.3 a	3.12 b	8.4 a	6.7 a	111 ab
GLD_S++	0.2 b	19.4 b	3.25 a	7.8 a	6.1 a	66 b
**2017**						
Symptomless	0.6 a	22.5 a	3.23 a	8.7 a	6.5 a	245 a
GLD_S+	0.3 b	22.4 a	3.23 a	8.8 a	6.5 a	178 b
GLD_S++	n.a.	n.a.	n.a.	n.a.	n.a.	n.a.

## Data Availability

The data used publicly available data: National Center for Biotechnology Information (NCBI) under the reference PRJNA594635.And produced the following Genbank accessions: ON221453 to ON221466; ON237610; ON221467; ON221468; ON583999.

## Supplementary Materials

The following supporting information can be downloaded at: https://www.mdpi.com/article/10.3390/v14061333/s1. Table S1. (A) Results of RT-PCR detection of the 11 viruses detected in this study on individual plants of the three treatments (Symptomless, GLD_S+ and GLD_S++) grafted onto the 5BB rootstock. Only eight plants were available for the Symptomless treatment. (B) PCR primers and conditions used (and reference). Refs. [58,59,60,61,62,63,64,65,66] cited in Supplementary. Table S2. Determination of the virome of each treatment in the experiment. Total number of reads mapping, percentage coverage and mean coverage on the different virus/viroid identified obtained by mapping using Geneious v11.1 with the Medium Low default parameter with 2 iterations (except for GFkV where 1 iteration was used). GenBank accession added for the sequences deposited. Total number of read mapped and sequenced are listed at the bottom with the calculation of the number of viral reads mapped per million reads sequenced. Virus acronyms: GRSPaV—grapevine rupestris stem pitting-associated virus; GRGV—grapevine red globe virus; GAMaV—grapevine asteroid mosaic-associated virus; GSyV-1—grapevine Syrah virus 1; GFkV—grapevine fleck virus; GPGV-grapevine Pinot gris virus; GLRaV-1—grapevine leafroll-associated virus 1; GLRaV—3-grapevine leafroll-associated virus 3; GRVFV—grapevine rupestris vein feathering virus; GVT—grapevine virus T; GVB—grapevine virus B.
