# Supplementary material for: Nuances of Responses to Two Sources of Grapevine Leafroll Disease on Pinot Noir Grown in the Field for 17 Years
Source: Viruses. 2022 Jun 18;14(6):1333. doi: 10.3390/v14061333 (PMC9227476; doi:10.3390/v14061333)
Supplement: Supplementary file 1 [file viruses-14-01333-s001.zip › viruses-1764462 Supplementary corrected.pdf]

Supplementary Table S1.
Top: Results of RT-PCR detection of the 11 viruses detected in this study on individual plants of the three treatments (Symptomless, GLD_S+ and GLD_S++) grafted onto the 5BB rootstock. Only eight plants were available for the Symptomless treatment.
Bottom: PCR primers and conditions used (and reference)

	Closteroviridae		*Betaflexiviridae*				*Tymoviridae*				
	**GLRaV-1**	**GLRaV-3**	**GVB**	**GPGV**	**GRSPaV**	**GVT**	**GRGV**	**GFkV**	**GAMaV**	**GSyV-1**	**GRVFV**
Healthy 5BB_01	-	-	-	-	+	-	+	-	-	+	-
Healthy 5BB_02	-	-	-	-	+	-	+	-	+	+	-
Healthy 5BB_03	-	-	-	-	+	-	+	-	-	+	-
Healthy 5BB_04	-	-	-	-	+	-	+	-	-	-	-
Healthy 5BB_05	-	-	-	-	+	-	-	-	-	-	-
Healthy 5BB_07	-	-	-	-	+	-	+	-	-	+	-
Healthy 5BB_08	-	-	-	-	+	-	-	-	-	+	-
Healthy 5BB_10	-	-	-	-	+	-	-	-	+	+	-
GLD_S+ 5BB_01	+	-	-	+	+	-	+	+	-	-	+
GLD_S+ 5BB_02	+	-	-	-	+	-	+	+	-	-	+
GLD_S+ 5BB_03	+	-	-	+	+	-	+	+	-	-	+
GLD_S+ 5BB_04	+	-	-	-	+	-	+	+	-	-	+
GLD_S+ 5BB_05	+	-	-	-	+	-	+	+	-	-	+
GLD_S+ 5BB_06	+	-	-	-	+	-	+	+	-	-	+
GLD_S+ 5BB_07	+	-	-	-	+	-	+	+	-	-	+
GLD_S+ 5BB_08	+	-	-	-	+	-	+	+	-	-	+
GLD_S+ 5BB_09	+	-	-	-	+	-	+	+	-	-	+
GLD_S+ 5BB_10	+	-	-	-	+	-	+	-	-	-	+
GLD_S++ 5BB_01	+	+	+	+	+	+	+	-	-	-	+
GLD_S++ 5BB_02	+	+	+	-	+	+	-	-	-	-	+
GLD_S++ 5BB_03	+	+	+	-	+	+	+	-	-	-	+
GLD_S++ 5BB_04	+	+	+	-	+	+	+	-	-	-	+
GLD_S++ 5BB_05	+	+	+	-	+	+	+	-	-	-	+
GLD_S++ 5BB_06	+	+	+	+	+	+	-	-	-	-	+
GLD_S++ 5BB_07	+	+	+	-	+	+	+	-	-	-	+
GLD_S++ 5BB_08	+	+	+	-	+	+	+	-	-	-	+
GLD_S++ 5BB_09	+	+	+	-	+	+	+	-	-	-	+
GLD_S++ 5BB_10	+	+	+	-	+	+	+	-	-	-	+

**Virus name**	**Acronym**	**Primer name**	**Forward primer sequence (5'-3')**	**Amplicon size**	**Annealing temperature**	**Reference**
Grapevine leafroll-associated virus 1	GLRaV-1	LQV1-H47<br>LEVI-C447	GTTACGGCCCTTTGTTTATTATGG<br>CGACCCCTTTATTGTTTGAGTATG	401bp	60°C	[58]
Grapevine leafroll-associated virus 3	GLRaV-3	LR3POLF1<br>LR3POLR1	ACGTAACGGGGCAGAATATAGT<br>TATCAACACCAAGTGTCAAGAGTA	282bp	60°C	[59]
Grapevine virus B	GVB	GB1<br>GB2	GTGCTAAGAACGTCTTCACAGC<br>ATCAGCAAACACGCTTGAACCG	450bp	65°C	[60]
Grapevine Pinot gris virus	GPGV	GPGV RepF<br>GPGV RepR	TGAGGCATTCGATGTTTCCCA<br>ACCCAATCAAGCCATGAACCT	525bp	60°C	[61]
Grapevine rupestris stem pitting-assocaited virus	GRSPaV	RSP35<br>RSP36	AGRYTTAGRGTRGCTAARGC<br>CACATRTCATCVCCYGCAAA	478bp	55°C	[62]
Grapevine virus T	GVT	GVT-8123F<br>GVT-8434R	AGGTTYTGCATGTATTACTC<br>TTGTAAGCTGGAGCACCCAT	312bp	54°C	This study
Grapevine redglobe virus	GRGV	RG-CF-F1<br>RG-CF-R1	GAATTCGCTGTCGGCCACTC<br>AGTGAGAGGAGAGATTCCATC	453bp	60°C	[63]
Grapevine fleck virus	GFkV	GFkV6445F<br>GFkV6964R	CCGTCTGCTGACCAGCCTG<br>CGACGCAGGCGTCATTGCG	520bp	55°C	[64]
Grapevine asteroid mosaic-associated virus	GAMaV	6165F<br>6631R	CTCGCGCTCCTCGCATTGTT<br>CGTGACGAGGTTGGTCCCA	467bp	59°C	[65]
Grapevine Syrah virus 1	GSyV-1	Det-F<br>Det-R	CAAGCCATCCGTGCATCTGG<br>GCCGATTTGGAACCCGATGG	296bp	60°C	[66]
Grapevine rupestris vein feathering virus	GRVFV	GRVFV_6165F<br>GRVFV_6600R	ACTCWCYATCCCCTTCCAGT<br>GCTGACCATGCCACGAATCA	445bp	60°C	This study

Supplementary Table S2. Determination of the virome of each treatment in the experiment. Total number of reads mapping, percentage coverage and mean coverage on the different virus/viroid identified obtained by mapping using Geneious v11.1 with the Medium Low default parameter with 2 iterations (except for GFkV where 1 iteration was used). GenBank accession added for the sequences deposited. Total number of read mapped and sequenced are listed at the bottom with the calculation of the number of viral reads mapped per million reads sequenced. Virus acronyms: GRSPaV- grapevine rupestris stem pitting-associated virus; GRGV - grapevine red globe virus; GAMaV- grapevine asteroid mosaic-associated virus; GSyV-1 - grapevine Syrah virus 1; GFkV - grapevine fleck virus; GPGV - grapevine Pinot gris virus; GLRaV-1- grapevine leafroll-associated virus 1; GLRaV-3 - grapevine leafroll-associated virus 3; GRVFV - grapevine rupestris vein feathering virus; GVT - grapevine virus T; GVB - grapevine virus B.

	HEALTHY				GLD_S+				GLD_S++			
	reads mapped	% coverage	Mean Coverage	GenBank #	reads mapped	% coverage	Mean Coverage	GenBank #	reads mapped	% coverage	Mean Coverage	GenBank #
GRSPaV_I	228 868	100%	1 912	ON221452	156 605	100%	1 330	ON221454	275 809	100%	2 278	ON221457
GRSPaV_II	79 728	100%	671	ON221450	116 761	96.8%	998	ON221453	190 574	96.5%	1 619	ON221456
GRSPaV_III	223 636	99.7%	1 898	ON221451	246 174	99.8%	2 100	ON221455	348 787	100%	3 079	ON221458
GVT									66 455	100%	571	ON221461
GPGV					18 589	100.0%	189	ON237610				
GVB #1									57 372	100%	569	ON221459
GVB #2									1 506 248	99.9%	14 868	ON221460
GLRaV-3									856	91.9%	3	
GLRaV-1					3 073	99.6%	12	ON221467	1 430	94.2%	6	ON221468
GAMaV	10 327	87.8%	115	ON583999								
GFkV					409 699	96.9%	4 146	ON221466				
GRGV	340	75.7%	4		3 588	96.1%	38	ON221462	40 353	100%	443	ON221463
GSyV-1	2 263	98.4%	26	ON221449								
GRVFV					7 142	99.9%	80	ON221464	12 496	100%	149	ON221465
HSVd	3 502	100%	836		3 019	100%	700		5 495	100%	1 274	
GYSVd	7 630	100%	1 427		6 971	100%	1 299		7 056	100%	1 312	
Total mapped	*556 294*				*971 621*				*2 512 931*			
Sequenced (M)	*160.808*				*145.387*				*136.789*			
Viral read per M	*3 459*				*6 683*				*18 371*			

References

58. Osman, F.; Rowhani, A., Application of a spotting sample preparation technique for the detection of pathogens in woody plants by RT-PCR and real-time PCR (TaqMan). Journal of Virological Methods 2006, 133, (2), 130-136.

59. Beuve, M.; Moury, B.; Spilmont, A.-S.; Sempé-Ignatovic, L.; Hemmer, C.; Lemaire, O., Viral sanitary status of declining grapevine Syrah clones and genetic diversity of Grapevine Rupestris stem pitting-associated virus. European Journal of Plant Pathology 2013, 135, (2), 439-452.

60. Levy, L.; Lee, M.; Hadidi, A., Simple and rapid preparation of infected plant tissue extracts for PCR amplification of virus, viroid, and MLO nucleic acids. Journal of Virological Methods 1994, 49, (3), 295-304.

61. Saldarelli, P.; Giampetruzzi, A.; Morelli, M.; Malossini, U.; Pirolo, C.; Bianchedi, P.; Gualandri, V., Genetic variability of Grapevine Pinot gris virus and its association with grapevine leaf mottling and deformation. Phytopathology 2015, 105, (4), 555-563.

62. Terlizzi, F.; Li, C.; Ratti, C.; Qiu, W.; Credi, R.; Meng, B., Detection of multiple sequence variants of Grapevine rupestris stem pitting-associated virus using primers targeting the polymerase domain and partial genome sequencing of a novel variant. Annals of Applied Biology 2011, 159, (3), 478-490.

63. Beuve, M.; Candresse, T.; Tannières, M.; Lemaire, O., First report of grapevine redglobe virus (GRGV) in grapevine in France. Plant Disease 2015, 99, (3), 422-422.

64. Glasa, M.; Predajňa, L.; Komínek, P., Grapevine fleck virus isolates split into two distinct molecular groups. Journal of Phy-topathology 2011, 159, (11-12), 805-807.

65. Xiao, H.; Meng, B., First report of Grapevine asteroid mosaic-associated virus and Grapevine rupestris vein feathering virus in Grapevines in Canada. Plant Disease 2016, 100, (10), 2175-2175.

66. Al Rwahnih, M.; Daubert, S.; Golino, D.; Rowhani, A., Deep sequencing analysis of RNAs from a grapevine showing Syrah decline symptoms reveals a multiple virus infection that includes a novel virus. Virology 2009, 387, (2), 395-401.
